# A Ação da Bebida Energética na Frequência Cardíaca de Recuperação Independe da Capacidade Funcional

**DOI:** 10.36660/abc.20220596

**Published:** 2022-10-05

**Authors:** Rodrigo Freire Dutra, Francisco Eberth Marinho Marques, Luciana Diniz Nagem Janot de Matos

**Affiliations:** 1 Hospital Israelita Albert Einstein São Paulo SP Brazil Hospital Israelita Albert Einstein , São Paulo , SP – Brazil

**Keywords:** Bebidas Energéticas, Suplementos Nutricionais, Sistema Nervoso Autônomo, Sistema Cardiovascular, Exercício

O consumo de bebidas energéticas (BE) cafeinadas tem aumentado de forma considerável nos últimos anos. ^1^ Aumento da capacidade de concentração, ganho de rendimento no trabalho e aumento de performance na atividade física são algumas das razões que levam a busca da bebida. ^2^

Durante a pandemia, com as mudanças na rotina impostas pela necessidade de isolamento social, relatou-se um aumento na ingesta de BE em alguns trabalhos. Houve também maior consumo na população jovem motivada por fatores como melhor desempenho em esportes e capacidade de concentração. ^3 , 4^

Doses de cafeína de até 400 mg/dia ou até 200 mg em dose única são consideradas seguras do ponto de vista cardiovascular. ^5^ No entanto, com frequência adicionam-se substâncias como taurina, guaraná, vitaminas e minerais que podem potencializar o efeito da BE e, consequentemente, aumentar o risco de eventos adversos. ^6^

Fletcher et al., ^7^ publicaram em 2017 que a ingesta de 32 oz. (946 ml) de BE contendo 320 mg de cafeína levou a um aumento estatisticamente significativo do intervalo QTc e pressão arterial sistólica quando comparado a ingesta isolada de cafeína na mesma quantidade (320 mg). ^7^ Outros trabalhos mostraram eventos adversos como fibrilação atrial, fibrilação ventricular e elevação de segmento ST relacionados ao consumo da BE. ^8^

A magnitude da queda da frequência cardíaca (FC) no primeiro minuto da fase de recuperação pós teste de esforço físico reflete a capacidade de reativação do sistema nervoso autônomo parassimpático após exercício. Esse parâmetro é preditor importante de risco cardiovascular e de prognóstico. ^9^ A variabilidade da frequência cardíaca (VFC) também é um importante meio de avaliação do funcionamento do sistema nervoso autônomo de forma não invasiva. Estudos anteriores analisaram o efeito da BE e da cafeína em relação à recuperação da FC e da VFC após exercício físico. Em alguns trabalhos utilizando 300-400 mg de cafeína antes do exercício houve um retardo na reativação parassimpática na fase de recuperação. ^10 , 11^ Tais achados, no entanto, ainda são divergentes na literatura. ^12^

Em trabalho anterior do grupo, Porto et al., ^13^ analisaram o efeito da BE antes da atividade física e não encontraram diferenças no controle autonômico da FC na fase de recuperação após exercício aeróbico submáximo. ^13^

Neste trabalho mais recente, Porto et al., ^14^ avaliaram o impacto da BE na VFC e na recuperação da FC após exercício em indivíduos com diferentes capacidades cardiorrespiratórias. Apesar de utilizar protocolo semelhante ao estudo anterior, desta vez o grupo encontrou impacto da BE tanto naqueles com alta como com baixa capacidade cardiorrespiratória. ^14^

Em relação à metodologia, consideramos que o trabalho apresenta pontos fortes que foram a utilização de um protocolo randomizado, *crossover* e duplo-cego, que contribuíram para a diminuição dos vieses. Um parâmetro importante, no entanto, que poderia ter sido analisado seria a FC no pico do esforço físico e a sua comparação com a FC ao final do primeiro minuto de recuperação. Esse indicador reforçaria os achados da VFC na avaliação da reativação vagal e seu fator prognóstico. ^9^

Importante relatar que o trabalho utilizou 250 ml de BE contendo 32 mg de cafeína. Esse volume está abaixo do descrito em trabalhos anteriores mostrando efeitos arritmogênicos da BE e com dose de cafeína bastante abaixo da máxima considerada segura. Atualmente existem diversos compostos energéticos solúveis e em cápsulas que utilizam doses de 100 a 200 mg de cafeína associados a outras substâncias potencializadoras do seu efeito.

Outro importante ponto a ser levantado é que a cafeína pode apresentar efeito de tolerância após uso prolongado. ^15^ Isso pode levar a um consumo maior e consequentemente maior potencial de efeitos colaterais ao longo do tempo.

A ingesta excessiva de BE pode provocar diversos efeitos adversos do ponto de vista cardiovascular. Seu consumo mais frequente, além do aumento no consumo em jovens merece atenção, principalmente quando associado a outras substâncias. Trabalhos futuros avaliando substâncias energéticas disponíveis em cápsulas ou solúveis contendo doses maiores de cafeína, seus efeitos em pessoas sedentárias e o impacto também em mulheres seriam de grande importância.
