# Supplementary material for: A Ação da Bebida Energética na Frequência Cardíaca de Recuperação Independe da Capacidade Funcional
Source: Arq Bras Cardiol. 2022 Oct 5;119(4):562–3. [Article in Portuguese] doi: 10.36660/abc.20220596 (PMC9563893; doi:10.36660/abc.20220596)
Supplement: Translation

# Translation: The Action of the Energy Drink on the Recovery Heart Rate is Independent of the Functional Capacity

**Authors:** Rodrigo Freire Dutra, Francisco Eberth Marinho Marques, Luciana Diniz Nagem Janot de Matos

**Affiliations:** 1 Brazil Hospital Israelita Albert Einstein, São Paulo, SP – Brazil

**Keywords:** Energy Drinks, Dietary Supplements, Autonomc Nervous System, Cardiovascular System, Exercise

The consumption of caffeinated energy drinks (ED) has increased considerably in recent years.^1^ Increased ability to concentrate, gain in work performance and increased performance in physical activity are some of the reasons that lead to the search for the drink.^2^

During the pandemic, with changes in routine imposed by the need for social isolation, some works reported an increase in ED intake. There was also greater consumption in the young population motivated by factors such as better performance in sports and the ability to concentrate.^3 , 4^

Caffeine doses of up to 400 mg/day or up to 200 mg in a single dose are considered safe from a cardiovascular point of view.^5^ However, substances such as taurine, guarana, vitamins, and minerals are often added that can potentiate the effect of the ED and, consequently, increase the risk of adverse events.^6^

Fletcher et al.^7^ published in 2017 that the intake of 32 oz. (946 ml) of an ED containing 320 mg of caffeine led to a statistically significant increase in QTc interval and systolic blood pressure when compared to caffeine intake alone in the same amount (320 mg).^7^ Other studies have shown adverse events such as atrial fibrillation, ventricular fibrillation and ST-segment elevation related to ED consumption.^8^

The magnitude of the heart rate (HR) drops in the first minute of the post-exercise test recovery phase reflects the ability of the parasympathetic autonomic nervous system to reactivate after exercise. This parameter is an important predictor of cardiovascular risk and prognosis.^9^ Heart rate variability (HRV) is also an important means of non-invasively assessing the functioning of the autonomic nervous system. Previous studies have analyzed the effect of ED and caffeine on HR and HRV recovery after physical exercise. In some studies using 300-400 mg of caffeine before exercise, there was a delay in parasympathetic reactivation in the recovery phase.^10 , 11^ Such findings, however, are still divergent in the literature.^12^

In previous work by the group, Porto et al.^13^ analyzed the effect of the ED before physical activity and found no differences in the autonomic control of HR in the recovery phase after submaximal aerobic exercise.^13^

In this most recent work, Porto et al.^14^ evaluated the impact of ED on HRV and HR recovery after exercise in individuals with different cardiorespiratory capacities. Despite using a protocol similar to the previous study, this time, the group found an impact of the ED in both those with high and low cardiorespiratory capacity.^14^

Regarding the methodology, we consider that the work has strong points, that were the use of a randomized, crossover and double-blind protocol, which contributed to the reduction of biases. An important parameter that could have been analyzed would be the HR at the peak of the physical effort and its comparison with the HR at the end of the first minute of recovery. This indicator would reinforce the HRV findings in the assessment of vagal reactivation and its prognostic factor.^9^

It is important to report that the study used a 250 ml ED containing 32 mg of caffeine. This volume is below that described in previous studies showing arrhythmogenic effects of the ED and with a dose of caffeine well below the maximum considered safe. Currently, several soluble energy compounds and capsules use doses of 100 to 200 mg of caffeine associated with other substances that enhance their effect.

Another important point is that caffeine may be tolerable after prolonged use.^15^ This can lead to higher consumption and, consequently, greater potential for side effects over time.

Excessive intake of ED can cause several adverse effects from a cardiovascular point of view. Its more frequent consumption, in addition to the increase in consumption in young people, deserves attention, especially when associated with other substances. Future work evaluating energy substances available in capsules or soluble containing higher doses of caffeine, their effects on sedentary people and the impact on women would be of great importance.
